# Neurobiological Alterations Induced by SARS-CoV-2: Insights from Variant-Specific Host Gene Expression Patterns in hACE2-Expressing Mice

**DOI:** 10.3390/v17030329

**Published:** 2025-02-27

**Authors:** Hamid Reza Jahantigh, Amany Elsharkawy, Anchala Guglani, Komal Arora, Lila D. Patterson, Mukesh Kumar

**Affiliations:** 1 Department of Biology, College of Arts and Sciences, Georgia State University, Atlanta, GA 30303, USA; hjahantigh@gsu.edu (H.R.J.); aelsharkawy2@student.gsu.edu (A.E.); aguglani@gsu.edu (A.G.); karora@gsu.edu (K.A.); lpatterson37@gsu.edu (L.D.P.); 2 Center of Diagnostics and Therapeutics, Georgia State University, Atlanta, GA 30303, USA

**Keywords:** SARS-CoV-2, neuroinflammation, variants of concern, omicron, RNA sequencing

## Abstract

Since the onset of the COVID-19 pandemic, various severe acute respiratory syndrome coronavirus-2 (SARS-CoV-2) variants have emerged. Although the primary site of SARS-CoV-2 infection is the lungs, it can also affect the brain and induce neurological symptoms. However, the specific effects of different variants on the brain remain unclear. In this study, a whole-transcriptome analysis was conducted using the brain tissues of K18-hACE2 mice infected with the ancestral B.1 (Wuhan) variant and with major SARS-CoV-2 variants of concern, including B.1.1.7 (Alpha), B.1.351 (Beta), B.1.617.2 (Delta) and B.1.529 (Omicron). After sequencing, differential gene expression, gene ontology (GO) and genome pathway enrichment analyses were performed. An Immune Cell Abundance Identifier (ImmuCellAI) was used to identify the abundance of different cell populations. Additionally, RT-qPCR was used to validate the RNA-seq data. The viral load and hierarchical clustering analyses divided the samples into two different clusters with notable differences in gene expression at day 6 post-infection for all variants compared to the control group. GO and the Kyoto Encyclopedia of genes and genomes enrichment analyses revealed similar patterns of pathway enrichment for different variants. ImmuCellAI revealed the changes in immune cell populations, including the decrease in CD4^+^ T and B cell proportions and the increase in CD8^+^ T and dendritic cell proportions. A co-expression network analysis revealed that some genes, such as *STAT1*, interleukin-*6* (*IL-6*) and tumor necrosis factor alpha (*TNF-α*), were dysregulated in all variants. A RT-qPCR analysis for *IL-6*, *CXCL10* and *IRF7* further validated the RNA-seq analysis. In conclusion, this study provides, for the first time, an extensive transcriptome analysis of a K18-hACE2 mouse brain after infection with major SARS-CoV-2 variants.

## 1. Introduction

Severe acute respiratory syndrome-coronavirus-2 (SARS-CoV-2) has infected approximately 700 million people since 2019, posing a serious threat to public health worldwide [1]. Although different vaccines have been developed against SARS-CoV-2, their efficacy has decreased as new variants of concern (VOCs) have emerged [2,3,4].

SARS-CoV-2 mainly affects the respiratory system [5,6], and most patients recover from acute infection. However, some patients experience mild to severe neurological symptoms. The central nervous system (CNS) is affected by SARS-CoV-2 in approximately 0.04–0.20% of all cases [7,8]. Neurological complications, such as brain fog, loss of taste and smell, changed mental status and anosmia, have been reported in COVID-19 patients [9]. Several reports suggest that COVID-19 neuropathology results from inflammation and hypoxia rather than from direct viral invasion of the CNS [10,11]. However, COVID-19 symptoms differ based on the variant and rate of immunization prior to infection [12]. Emerging VOCs display differential pathogenicity in humans and in animal models [8]. For instance, the Omicron variant causes relatively fewer pulmonary lesions, lower viral titers and milder pneumonia in the lungs than the original Wuhan or Delta variants in COVID-19 disease models [13,14].

SARS-CoV-2 has four structural proteins, namely a spike (S) protein, an envelope (E) protein, a membrane (M) glycoprotein and a nucleocapsid (N) protein. The S protein, which is a class I fusion protein, mediates viral attachment to the host cell membrane through the functional receptor, human angiotensin-converting enzyme 2 (hACE2) [15]. Transgenic mice expressing hACE2 under the control of the cytokeratin 18 promoter (K18-hACE2) have been developed to study the pathogenesis of SARS-CoV-2 infection in animal models [16,17]. K18-hACE2 mice intranasally infected with SARS-CoV-2 exhibit alterations in the brain [17,18]. This spread to the brain induces the expression of several proinflammatory cytokines and chemokines [19,20]. However, the neurotropism of VOCs remains largely unclear [17,20].

To better understand the pathogenesis of different SARS-CoV-2 variants in the brain, we intranasally infected K18-hACE mice with major SARS-CoV-2 variants and conducted a whole-transcriptome analysis of the brain tissues.

## 2. Materials and Methods

### 2.1. In Vivo Animal Experiments

K18-hACE2 mice (2B6. Cg-Tg (K18-ACE2)2Prlmn/J) were purchased from the Jackson Laboratory. The Institutional Animal Care and Use Committee of Georgia State University approved all experiments (approval protocol number: A24003). K18-hACE2 mice were intranasally infected with phosphate-buffered saline (mock) or 10^4^ plaque-forming units (PFU) of B.1 (Wuhan; BEI# NR-52281), B.1.1.7 (Alpha; BEI# NR-54000), B.1.351 (Beta; BEI# NR-54008), B.1.617.2 (Delta; Northwestern Reference Laboratory, Clinical Isolate #2333067) and B.1.1.529 (Omicron; BEI# NR-56461) [3]. On day 6 post-infection, mice (*n* = 4 per group) were anesthetized with isoflurane and perfused with 1× PBS, with brain samples being obtained and flash-frozen with 2-methylbutane (Sigma, St. Louis, MO, USA) for subsequent analysis [3]. An equal number of male and female mice were used for each group.

### 2.2. Reverse Transcription-Polymerase Chain Reaction (RT-PCR)

Brain samples (*n* = 4 per group) from the mock- and SARS-CoV-2-infected animals were homogenized in RLT buffer (Thermo Fisher Scientific, Waltham, MA, USA). Total RNA was extracted using the Qiagen RNeasy Mini Kit (Qiagen, Germantown, MD, USA), following the manufacturer’s protocol. RNA quality and concentration were determined by measuring the A260/280 and A260/230 absorbance ratios using the NanoDrop spectrophotometer (Thermo Fisher Scientific). Subsequently, 1000 ng of RNA was reverse transcribed into cDNA using the Bio-Rad iScript cDNA Synthesis Kit (Catalogue #1708891, Hercules, CA, USA). PCR was performed with 2 µL of the resulting cDNA diluted in RNase-free water. Gene expression levels were determined using the SsoAdvanced Universal SYBR Green Supermix (Catalogue #1725271; Bio-Rad, USA). Gene expression was normalized to that of glyceraldehyde 3-phosphate dehydrogenase, and the fold change was calculated. Viral genome copies were determined using the SsoAdvanced Universal Probes Supermix (Catalogue #1725284; Bio-Rad) and SARS-CoV-2 N primers from Integrated DNA Technologies (Catalogue #10006713, Coralville, IA, USA). The primer sequences used for RT-qPCR are listed in Table 1 [3].

### 2.3. Transcriptome Profiling Analysis

Brain samples (*n* = 4 per group) from the mock- and SARS-CoV-2-infected animals were used for transcriptome profiling analyses of the differentially expressed genes (DEGs) [21,22]. The RNA integrity number (RIN) > 7.0 on the 2100 Bioanalyzer (Agilent Technologies, Inc., Santa Clara, CA, USA) indicated the integrity of the total RNA. A library was constructed using the TruSeq Stranded mRNA Sample Preparation Guide and TruSeq Stranded mRNA LT Sample Prep Kit. Finally, transcriptome profiling was performed using the Illumina platform.

The Phred quality score in each cycle determined the quality of the generated data. Adapter sequences and bases with scores <3 were eliminated using the Trimmomatic program [23,24]. Reads shorter than 36 bp were removed to create trimmed data. UCSC mm10 was used as the reference genome to map the cDNA fragments acquired from RNA sequencing. Using StringTie, the known genes and transcripts were used to construct a reference genome model. The read count and normalized value of fragments per kilobase of transcript per million mapped reads (FPKM) were used to determine the gene expression in each sample. A differential expression analysis was performed using the Limma Voom package in Galaxy Portal [25,26]. The false discovery rate was determined using the Benjamini–Hochberg correction method with q-value < 0.05. Fold-change > 2 indicated upregulated DEGs, whereas fold-change <0.5 indicated downregulated DEGs. Additionally, the “cluster profiler” and “KEGGREST” packages were used for gene enrichment analyses and “heatmaps”, “ggplot2”, and “ggcorrpolt” were used to visualize the results.

### 2.4. Immune Cell Infiltration and Protein–Protein Interaction (PPI) Analysis

An Immune Cell Abundance Identifier (ImmuCellAI) uses a gene expression dataset to determine the proportions of 24 immune cells [27,28], including 18 T cell subtypes and six other immune cell types. The extent of immune cell infiltration was determined by calculating the differences in the abundances of infiltrating immune cells between brain samples infected with different variants and mock-infected controls using the ImmuCellAI tool. R packages “ggplot2” and “ggcorrpolt” were utilized to generate boxplots and dot plots [29]. In addition, a PPI network was constructed using the Cytoscape 3.10.3 software [30]. Clusters and hub genes were identified using the MCODE and CytoHubba plug-ins, respectively [31,32].

### 2.5. Statistical Analyses

Statistical analyses were conducted using GraphPad Prism version 10 software. Statistical significance was set at * *p* < 0.05, ** *p* < 0.01, and *** *p* < 0.001.

## 3. Results and Discussion

### 3.1. Brain Viral Load After Infection with VOCs

**Figure 1:**
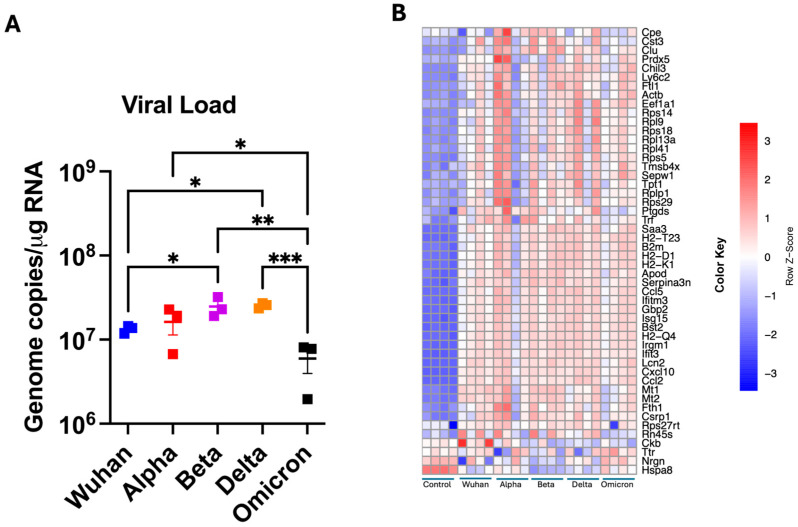
Viral load and DEGs analysis in the brain. (**A**) Viral load in the brain was determined via RT-PCR. Statistical significance was determined by one-way ANOVA, followed by a Dunn’s test (* *p* < 0.05; ** *p* < 0.01; *** *p* < 0.001. Bar represents the mean ± SEM (*n* = 3 per group). (**B**) Heatmap of the top 50 DEGs with the smallest *p* values at day 6 post-infection for individual samples (*n* = 4 per group). The color represents the level of expression based on the raw Z-score: the redder the color is, the greater the gene expression, while the blueness of the color is related to lower gene expression.

The viral load in the infected brains was assessed in all infection groups (Figure 1A). The results demonstrated the presence of SARS-CoV-2 RNA in the brain tissue at day 6 post-infection across all tested groups. Notably, viral RNA levels in Omicron-infected brains were lower compared to mice infected with other VOCs despite identical inoculum dosage. Viral RNA levels in Alpha-infected brains were comparable to Wuhan-infected brains. Compared to Wuhan-infected lungs, a significantly higher viral load was detected in Beta- and Delta-infected brains.

### 3.2. Dynamics of VOCs Pathogenesis in the Brain Determined via Hierarchical Clustering

Each library contained over 40 million raw and clean reads. The GC content was 46.59–52.45%, with the base percentages of Q20 > 97.51% and Q30 > 92.97%. Therefore, sequencing data were suitable for subsequent analyses (Supplementary Tables S1–S5). We performed hierarchical clustering to understand the dynamics of SARS-CoV-2 variants in the brain. Among the top 50 genes, several are known to be important for the pathogenesis of the SARS-CoV-2 infection (Figure 1B). For example, upregulated genes, such as CCL5, *CXCL10*, *CCL2* and *IFIT3*, play an important role in the inflammatory response against SARS-CoV-2 infection [33,34]. Also, significant changes were observed in genes like Lipocalin 2 (*LCN2*), Serpin Family A Member 3 (*SERPINA3*), Heat Shock Protein Family A Member 8 (*HSPA8*), Neurogranin (*NRGN*) and Peroxiredoxin 5 (*PRDX5*). These genes are important for oxidative stress and neurological dysregulation in the brain [35,36,37,38]. In addition, metallothionein 1 (*MT1*) and metallothionein 2 (*MT2*) exhibited significantly higher expression in the infected brains. Overall, this upregulation suggests a robust inflammatory and oxidative stress response to SARS-CoV-2 infection in the brain [39,40].

### 3.3. DEGs and Pathway Enrichment Analyses Revealed Variant-Specific Responses in the Brain

**Figure 2:**
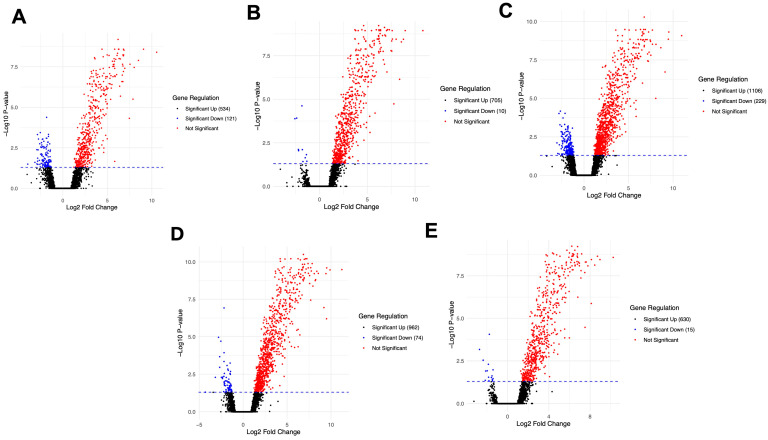
Volcano plots of DEGs in the brain at day 6 post-infection. (**A**) Wuhan vs. control, (**B**) Alpha vs. control, (**C**) Beta vs. control, (**D**) Delta vs. control and (**E**) Omicron vs. control.

DEG analysis results are shown in Figure 2A–E in which each point on the graph represents a specific gene or transcript. Red points indicate the significantly upregulated genes, blue points indicate the significantly downregulated genes, and black points indicate the genes with non-significant differences.

In the Wuhan-infected animals, 655 DEGs, comprising 534 upregulated and 121 downregulated genes, were observed (Figure 2A). Analysis of the Alpha-infected revealed 715 DEGs, with 705 upregulated and 10 downregulated genes (Figure 2B). The highest gene modulation was observed in the Beta-infected animals, with 1335 DEGs, including 1106 upregulated and 229 downregulated genes (Figure 2C). A total of 1036 DEGs were differentially expressed between the Delta-infected brains, with 962 upregulated and 74 downregulated genes (Figure 2D). Finally, total of 645 DEGs were found in the Omicron-infected brain, 630 of which were upregulated and 15 were downregulated (Figure 2E). Upregulated genes in all the infected groups were primarily related to the interferon and inflammatory response pathways, such as *IRF7*, *TAP1*, *IGTP*, *CXCL10*, *CCL5* and *SAA3*. Table 2 and Table 3 show the top ten upregulated and downregulated genes across different variants, respectively.

GO enrichment was performed to further understand the functional roles of the DEGs. The results for the top 30 significant pathways (*p* < 0.05) are presented in Supplementary Figure S1. In summary, the trends between variants were similar, with the enrichment of some notable pathways, such as positive regulation of protein phosphorylation, regulation of cellular catabolic processes, positive regulation of cell death, negative regulation of cell population proliferation, positive regulation of apoptotic processes, regulation of the MAPK cascade, cellular response to cytokine stimuli, inflammatory response, regulation of cytokine production and innate immune response.

**Figure 3:**
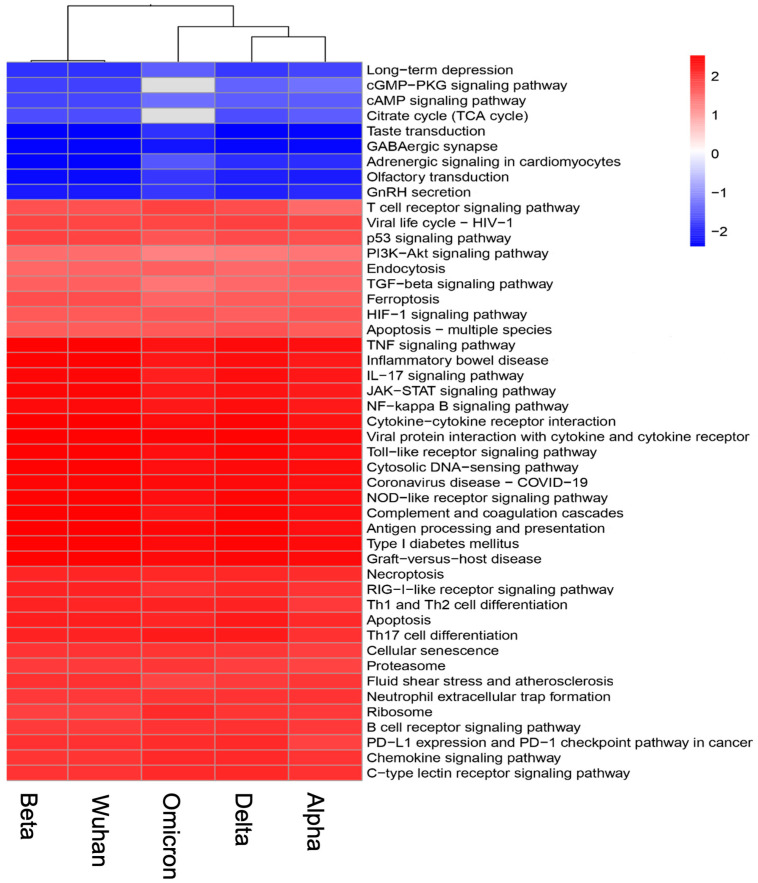
Enriched pathway analysis of the brain following infection with the Wuhan, Alpha, Beta, Delta and Omicron variants. An analysis was performed by comparing the enrichment of different pathways based on the normalized enrichment score (NES).

A Kyoto Encyclopedia of Genes and Genomes enrichment analysis was performed to analyze the differences among the variants (Figure 3). Consistent with the GO results, KEGG enrichment revealed that 103 different pathways were enriched for each variant, with many being common across the variants. These included downregulated pathways, such as gonadotropin-releasing hormone secretion, GABAergic synapse and the tricarboxylic acid cycle pathways, as well as upregulated pathways, such as the JAK-STAT signaling, cytokine and chemokine responses, HIF-1a, toll-like receptor signaling, the NOD-like receptor signaling, RIG-I-like receptor signaling, TNF signaling, apoptosis and cellular senescence pathways.

**Figure 4:**
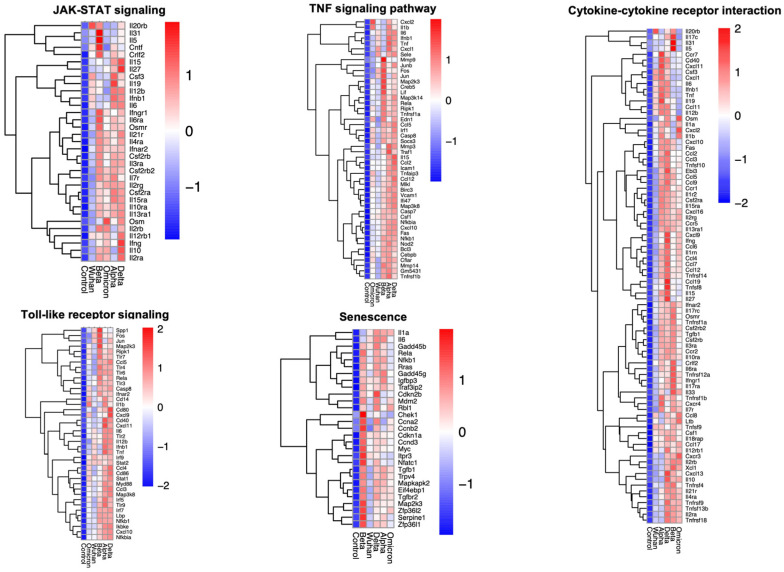
Mean gene expression of the most significantly expressed genes related to the Janus kinase-signal transducer and activator of transcription signaling (JAK-STAT), TNF signaling, toll-like receptors, cellular senescence and cytokine–cytokine receptor pathways.

To understand the similarities among the enriched pathways and determine the different genes related to the enriched pathways, a heatmap of the leading genes involved in cytokine–receptor interactions, TNF, JAK-STAT, cellular senescence, and toll-like receptors, is shown in Figure 4. Although the expression patterns of the genes related to different pathways were similar, some unique differences were observed. The Beta variant potently upregulated genes to a higher degree compared to the other variants. All variants induced cytokine and chemokine responses, but some notable differences were observed. For instance, compared to the Delta and Beta variants, Wuhan induced a lower cytokine response. These observations are consistent with previous reports that showed the Delta and Beta variants lead to increased organ lesions and disease pathology [41]. Also, the study results showed that different variants of SARS-CoV-2 could induce cellular senescence. Infection with different variants upregulated genes like cyclin-dependent kinase inhibitor 1A (*CDKN1A*) and cyclin-dependent kinase inhibitor 2B (*CDKN2B*), which play a role in cellular senescence [42,43]. Similarly, SARS-CoV-2 infection also induced the expression of genes like growth arrest and DNA damage-inducible beta (*GADD45B*), growth arrest and DNA damage-inducible gamma (*GADD45G*) and RELA, which play a key role in cellular stress, DNA damage response and keeping the senescence-associated secretory phenotype [44,45,46].

### 3.4. Impacts of VOCs on Immune Cell Infiltration Dynamics

**Figure 5:**
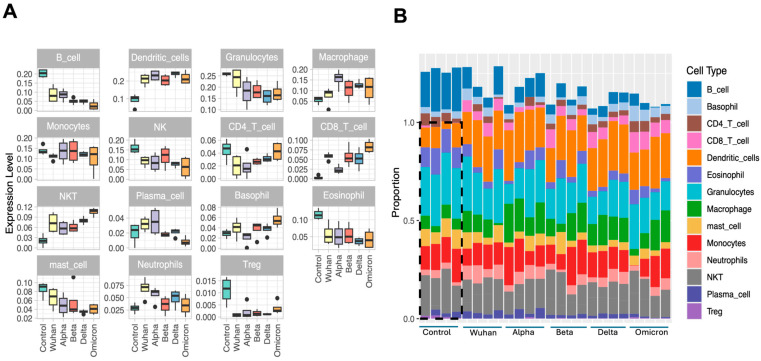
Degree of immune cell infiltration in the brains of the control and different variant-infected groups determined via an ImmuCellAI. (**A**) Boxplot showing the fraction of each cell type in each group. (**B**) Proportions of different cell fractions in the control and all infected groups.

In this study, ImmuCellAI algorithm was used to estimate immune infiltration in the brain across different variants. Significant differences in immune cell infiltration were observed between the infected and control groups. As shown in Figure 5A,B, similar trends in immune cell infiltration were observed for different variants. Fractions of CD8^+^ T cells, dendritic cells and macrophages were increased in all variant-infected groups compared to those in the control group. Conversely, fractions of CD4^+^ T cells, B cell subtypes and eosinophils were decreased in all variant-infected groups. It is known that alterations in different subsets of immune cells can affect the severity of COVID-19 disease [47,48]. For instance, CD4^+^ and CD8^+^ T cell counts are inversely correlated with patient survival [49,50]. Among all the changes in different immune cell subsets, an increase in CD8^+^ T cell fraction was significant for all variants and a decrease in B cell fraction was significant for all variants. Furthermore, significant changes were observed in the NK cell fractions for Wuhan-, Alpha-, Delta- and Omicron-infected brains. Infection with the Omicron variant decreased, whereas the other variants increased the neutrophil fraction. Additionally, all variants except the Wuhan and the Beta variant decreased the number of M2 macrophages.

### 3.5. Elucidation of the Shared and Unique Molecular Signatures of VOCs via a DEGs Network Analysis

**Figure 6:**
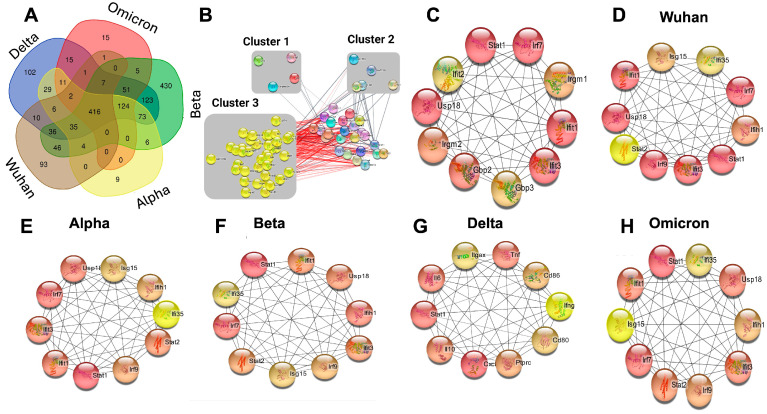
Protein–protein interaction (PPI) network analysis and hub gene identification. (**A**) A Venn diagram showing the overlap among the DEGs across all variants. (**B**) PPI network showing 76 overlapping genes, with three distinct clusters identified using MCODE. (**C**) Top 10 hub genes from the overlapping DEGs identified using the CytoHubba plugin. (**D**–**H**) Network diagrams showing the top 10 hub genes for different variants.

A comparison of DEGs revealed that 416 genes were shared among all variants (Figure 6A). Unique gene expression patterns specific to each variant were also observed. Genes that exhibited variant-specific alterations included 93 genes in the Wuhan variant, nine genes in the Alpha variant, 430 genes in the Beta variant, 102 genes in the Delta variant, and 15 genes in the Omicron variant (Figure 6A). Next, shared genes were subjected to network analyses and visualized using Cytoscape (Figure 6B). As shown in Figure 6B, three distinct clusters were identified using the MCODE plugin. Figure 6C shows the top 10 hub genes identified using the MCC algorithm and CytoHubba plugin. These genes were the interferon regulatory factor (*IRF*)-*7*, *STAT1*, *IFIT2*, *IRGM1*, *IFIT1*, *GBP3*, *GBP2*, *IFIT3*, *USP18* and *IRGM2*. A CytoHubba plugin was also used to highlight the top 10 ranked hub genes for each variant (Figure 6D–H). We linked the most important genes for each variant to different pathways. For instance, GTP-binding genes like *GBP-2* and *-3* are correlated to heightened immune responses and severe outcomes related to different variants [51]. Furthermore, different variants share genes related to the interferon response. However, most genes were shared among the different variants, and hub genes were uniformly present in some variants.

### 3.6. Experimental Validation via RT-qPCR

**Figure 7:**
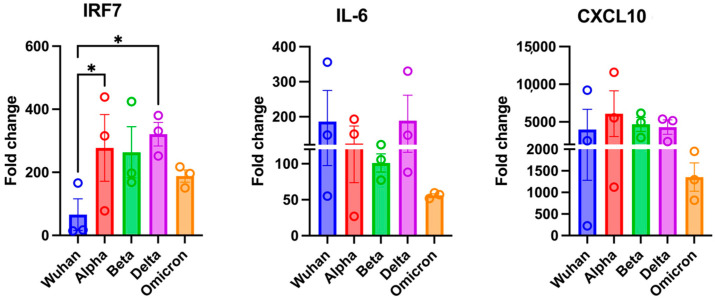
Validation of RNA-seq data. RT-qPCR of *IRF7*, *IL-6* and *CXCL10* in infected brains. A fold change in each gene was first normalized to the GAPDH gene, and a fold change in infected mice brains was calculated in comparison to corresponding control mice brains. A fold change is plotted for each mouse (*n* = 3 per group). Bar represents the mean ± SEM. Statistical significance was determined by one-way ANOVA, followed by a Dunn’s test (* *p* < 0.05).

To validate the RNA-seq results, we selected three DEGs from different pathways and performed RT-qPCR to analyze their average expression levels. Figure 7 shows that the expression patterns of *IRF7*, *IL-6*, and *CXCL10*. RT-qPCR results were consistent with the RNA-seq findings, confirming the reliability of the RNA-seq results.

## 4. Conclusions

This study provides, for the first time, an extensive transcriptome analysis of K18-hACE2 mice brains post-infection with major SARS-CoV-2 variants. Our results showed significant changes in immune-related pathways, such as toll-like receptor signaling, cytokine and chemokine receptor signaling, cellular senescence and apoptotic pathways. Interestingly, the Omicron variant had less immune activation than the pre-Omicron variants. This aligns with the epidemiological data, which indicates that the Omicron variant results in attenuated disease in humans. Additionally, there were significant increases in the number of CD8^+^ T cells, macrophages and dendritic cells in the brain, while the number of CD4^+^ T cells and B cell subtypes decreased across all variants. Importantly, DEGs and pathway analyses showed that infection with VOCs induced both similar and unique molecular fingerprints in the brain. Also, gene expression patterns unique to each variant showed patterns of gene expression that were shared between variants with key hub genes such as *IRF7*, *STAT1* and *GBP2*.

It is important to note that K18-hACE2 mice develop severe encephalitis upon SARS-CoV-2 infection, which is not commonly observed in humans. However, this mouse model is commonly used to study the pathogenesis of SARS-CoV-2 infection and to test the efficacy of anti-viral compounds and vaccines. In conclusion, this study is, to our knowledge, the first to examine transcriptome alterations in the K18-hACE2 mouse brain after infection with several major SARS-CoV-2 variants of concern.

## Figures and Tables

**Table 1:** Primer sequences used for RT-qPCR.

Gene (Accession No.)	Primer Sequence (5′–3′)
*IRF7* (NM_016850.3)	Forward: CCCCAGGATCATTTCTGGCA Reverse: AGGGTTCCTCGTAAACACGG
*CXC10* (NM_021274)	Forward: GGTCTGAGTCCTCGCTCAAG Reverse: GTCGCACCTCCACATAGCTT
*IL-6* (NM_000600)	Forward: CCAGGAGCCCAGCTATGAAC Reverse: CCCAGGGAGAAGGCAACTG

**Table 2:** Top 10 upregulated genes across different SARS-CoV-2 variants.

Wuhan	Alpha	Beta	Delta	Omicron
*CXCL10*	*CXCL10*	*CXCL10*	*CXCL10*	*CXCL10*
*LCN2*	*LCN2*	*LCN2*	*CCL5*	*CCL5*
*CCL2*	*CCL5*	*CCL5*	*LCN2*	*LCN2*
*CCL5*	*CCL2*	*SAA3*	*CHIL3*	*CCL2*
*SAA3*	*SAA3*	*CCL2*	*CCL2*	*ISG15*
*ISG15*	*ISG15*	*ISG15*	*SAA3*	*SAA33*
*CHIL3*	*CHIL3*	*CHIL3*	*ISG15*	*PLAC8*
*GBP2*	*GBP2*	*GBP2*	*CCL4*	*CCL4*
*IFIT3*	*RSAD2*	*IRF7*	*PLAC8*	*GBP2*
*IRF7*	*CCL12*	*PLAC8*	*CXCL9*	*CHIL3*

**Table 3:** Top 10 downregulated genes across different SARS-CoV-2 variants.

Wuhan	Alpha	Beta	Delta	Omicron
*DDN*	*GPR34*	*NPTXR*	*HCRT*	*GPR17*
*3110035E14RIK*	*GKN3*	*NEUROD6*	*NPTXR*	*UGT8A*
*KCNF1*	*FCRLS*	*UGT8A*	*GPR34*	*MYOC*
*TBR1*	*UGT8A*	*3110035E14RIK*	*KCNG1*	*GPR34*
*KCNG1*	*TRIB22*	*ADRA1D*	*FCRLs*	*GKN3*
*STX1A*	*MDGA1*	*GPR17*	*GKN3*	*PADI2*
*HPCAL4*	*NDST4*	*GPR34*	*MYOC*	*CHORDC1*
*NPTXR*	*TMEFF1*	*RGS4*	*BTBD17*	*TMEM229A*
*KCNV1*	*SLC30A10*	*MYL4*	*GPR17*	*TRIB2*
*RGS4*	*SLC2A5*	*KCNG1*	*MIR124A-1HG*	*ELOVL6*

## Data Availability

The original contributions presented in the study are included in the article/Supplementary Materials; further inquiries can be directed to the corresponding author.

## Supplementary Materials

The following supporting information can be downloaded at: https://www.mdpi.com/article/10.3390/v17030329/s1, Figure S1: GO enrichment analysis; Table S1: Wuhan Variant, Table S2: Alpha Variant, Table S3: Beta Variant, Table S4: Delta Variant, Table S5: Omicron Variant.

## References

[1] Firouzabadi N., Ghasemiyeh P., Moradishooli F., Mohammadi-Samani S. (2023). Update on the effectiveness of COVID-19 vaccines on different variants of SARS-CoV-2. Int. Immunopharmacol..

[2] Mohandas S., Jagannathan P., Henrich T.J., Sherif Z.A., Bime C., Quinlan E., Portman M.A., Gennaro M., Rehman J. (2023). Immune mechanisms underlying COVID-19 pathology and post-acute sequelae of SARS-CoV-2 infection (PASC). Elife.

[3] Natekar J.P., Pathak H., Stone S., Kumari P., Sharma S., Auroni T.T., Arora K., Rothan H.A., Kumar M. (2022). Differential pathogenesis of SARS-CoV-2 variants of concern in human ACE2-expressing mice. Viruses.

[4] Shao W., Chen X., Zheng C., Liu H., Wang G., Zhang B., Li Z., Zhang W. (2022). Effectiveness of COVID-19 vaccines against SARS-CoV-2 variants of concern in real-world: A literature review and meta-analysis. Emerg. Microbes Infect..

[5] Jin Y., Yang H., Ji W., Wu W., Chen S., Zhang W., Duan G. (2020). Virology, epidemiology, pathogenesis, and control of COVID-19. Viruses.

[6] Rothan H.A., Acharya A., Reid S.P., Kumar M., Byrareddy S.N. (2020). Molecular aspects of COVID-19 differential pathogenesis. Pathogens.

[7] Proust A., Queval C.J., Harvey R., Adams L., Bennett M., Wilkinson R.J. (2023). Differential effects of SARS-CoV-2 variants on central nervous system cells and blood–brain barrier functions. J. Neuroinflamm..

[8] Torabi S.H., Riahi S.M., Ebrahimzadeh A., Salmani F. (2023). Changes in symptoms and characteristics of COVID-19 patients across different variants: Two years study using neural network analysis. BMC Infect. Dis..

[9] Munblit D., Nicholson T.R., Needham D.M., Seylanova N., Parr C., Chen J., Kokorina A., Sigfrid L., Buonsenso D., Bhatnagar S. (2022). Studying the post-COVID-19 condition: Research challenges, strategies, and importance of Core Outcome Set development. BMC Med..

[10] Wenting A., Gruters A., van Os Y., Verstraeten S., Valentijn S., Ponds R., de Vugt M. (2020). COVID-19 neurological manifestations and underlying mechanisms: A scoping review. Front. Psychiatry.

[11] Almutairi M.M., Sivandzade F., Albekairi T.H., Alqahtani F., Cucullo L. (2021). Neuroinflammation and Its Impact on the Pathogenesis of COVID-19. Front. Med..

[12] Livieratos A., Gogos C., Akinosoglou K. (2024). Impact of Prior COVID-19 Immunization and/or Prior Infection on Immune Responses and Clinical Outcomes. Viruses.

[13] Wise J. (2022). Covid-19: Symptomatic infection with omicron variant is milder and shorter than with delta, study reports. BMJ Br. Med. J..

[14] Arabi M., Al-Najjar Y., Mhaimeed N., Salameh M.A., Paul P., AlAnni J., Abdelati A.A., Laswi I., Khanjar B., Al-Ali D. (2023). Severity of the Omicron SARS-CoV-2 variant compared with the previous lineages: A systematic review. J. Cell Mol. Med..

[15] Huang Y., Yang C., Xu X.-f., Xu W., Liu S.-w. (2020). Structural and functional properties of SARS-CoV-2 spike protein: Potential antivirus drug development for COVID-19. Acta Pharmacol. Sin..

[16] Jeong H., Woo Lee Y., Park I.H., Noh H., Kim S.-H., Kim J., Jeon D., Jang H.J., Oh J., On D. (2022). Comparison of the pathogenesis of SARS-CoV-2 infection in K18-hACE2 mouse and Syrian golden hamster models. Dis. Models Mech..

[17] Tarrés-Freixas F., Trinité B., Pons-Grífols A., Romero-Durana M., Riveira-Muñoz E., Ávila-Nieto C., Pérez M., Garcia-Vidal E., Perez-Zsolt D., Muñoz-Basagoiti J. (2022). Heterogeneous infectivity and pathogenesis of SARS-CoV-2 variants beta, delta and omicron in transgenic K18-hACE2 and wildtype mice. Front. Microbiol..

[18] Zheng J., Wong L.-Y.R., Li K., Verma A.K., Ortiz M.E., Wohlford-Lenane C., Leidinger M.R., Knudson C.M., Meyerholz D.K., McCray Jr P.B. (2021). COVID-19 treatments and pathogenesis including anosmia in K18-hACE2 mice. Nature.

[19] Aw Z.Q., Mok C.K., Wong Y.H., Chen H., Mak T.M., Lin R.T., Lye D.C., Tan K.S., Chu J.J.H. (2022). Early pathogenesis profiles across SARS-CoV-2 variants in K18-hACE2 mice revealed differential triggers of lung damages. Front. Immunol..

[20] Rothan H.A., Kumari P., Stone S., Natekar J.P., Arora K., Auroni T.T., Kumar M. (2022). SARS-CoV-2 infects primary neurons from human ACE2 expressing mice and upregulates genes involved in the inflammatory and necroptotic pathways. Pathogens.

[21] Kumar M., Belcaid M., Nerurkar V.R. (2016). Identification of host genes leading to West Nile virus encephalitis in mice brain using RNA-seq analysis. Sci. Rep..

[22] Iqbal N., Kumar P. (2022). Integrated COVID-19 Predictor: Differential expression analysis to reveal potential biomarkers and prediction of coronavirus using RNA-Seq profile data. Comput. Biol. Med..

[23] Bolger A.M., Lohse M., Usadel B. (2014). Trimmomatic: A flexible trimmer for Illumina sequence data. Bioinformatics.

[24] Pertea M., Pertea G.M., Antonescu C.M., Chang T.-C., Mendell J.T., Salzberg S.L. (2015). StringTie enables improved reconstruction of a transcriptome from RNA-seq reads. Nat. Biotechnol..

[25] Batut B., van den Beek M., Doyle M.A., Soranzo N. (2021). RNA-seq data analysis in galaxy. RNA Bioinform..

[26] Yang R.C., Huang K., Zhang H.P., Li L., Tan C., Chen H.C., Jin M.L., Wang X.R. (2022). Transcriptional landscape of human neuroblastoma cells in response to SARS-CoV-2. BMC Neurosci..

[27] Sun S., Guo W., Wang Z., Wang X., Zhang G., Zhang H., Li R., Gao Y., Qiu B., Tan F. (2020). Development and validation of an immune-related prognostic signature in lung adenocarcinoma. Cancer Med..

[28] Miao Y.-R., Xia M., Luo M., Luo T., Yang M., Guo A.-Y. (2022). ImmuCellAI-mouse: A tool for comprehensive prediction of mouse immune cell abundance and immune microenvironment depiction. Bioinformatics.

[29] Wickham H., Chang W., Wickham M.H. (2016). Package ‘ggplot2’. Create elegant data visualisations using the grammar of graphics. Version.

[30] Kohl M., Wiese S., Warscheid B. (2011). Cytoscape: Software for visualization and analysis of biological networks. Data Mining in Proteomics: From Standards to Applications.

[31] Cao L., Chen Y., Zhang M., Xu D.-q., Liu Y., Liu T., Liu S.-x., Wang P. (2018). Identification of hub genes and potential molecular mechanisms in gastric cancer by integrated bioinformatics analysis. PeerJ.

[32] Ma H., He Z., Chen J., Zhang X., Song P. (2021). Identifying of biomarkers associated with gastric cancer based on 11 topological analysis methods of CytoHubba. Sci. Rep..

[33] Rabaan A.A., Al-Ahmed S.H., Muhammad J., Khan A., Sule A.A., Tirupathi R., Mutair A.A., Alhumaid S., Al-Omari A., Dhawan M. (2021). Role of Inflammatory Cytokines in COVID-19 Patients: A Review on Molecular Mechanisms, Immune Functions, Immunopathology and Immunomodulatory Drugs to Counter Cytokine Storm. Vaccines.

[34] Khalil B.A., Elemam N.M., Maghazachi A.A. (2021). Chemokines and chemokine receptors during COVID-19 infection. Comput. Struct. Biotechnol. J..

[35] Suvarna K., Biswas D., Pai M.G.J., Acharjee A., Bankar R., Palanivel V., Salkar A., Verma A., Mukherjee A., Choudhury M. (2021). Proteomics and Machine Learning Approaches Reveal a Set of Prognostic Markers for COVID-19 Severity With Drug Repurposing Potential. Front. Physiol..

[36] Navhaya L.T., Blessing D.M., Yamkela M., Godlo S., Makhoba X.H. (2024). A comprehensive review of the interaction between COVID-19 spike proteins with mammalian small and major heat shock proteins. Biomol. Concepts.

[37] Sun B., Tang N., Peluso M.J., Iyer N.S., Torres L., Donatelli J.L., Munter S.E., Nixon C.C., Rutishauser R.L., Rodriguez-Barraquer I. (2021). Characterization and Biomarker Analyses of Post-COVID-19 Complications and Neurological Manifestations. Cells.

[38] Kanwal A., Zhang Z. (2024). Exploring common pathogenic association between Epstein Barr virus infection and long-COVID by integrating RNA-Seq and molecular dynamics simulations. Front. Immunol..

[39] Rice J.M., Zweifach A., Lynes M.A. (2016). Metallothionein regulates intracellular zinc signaling during CD4+ T cell activation. BMC Immunol..

[40] Dai H., Wang L., Li L., Huang Z., Ye L. (2021). Metallothionein 1: A New Spotlight on Inflammatory Diseases. Front. Immunol..

[41] Radvak P., Kwon H.-J., Kosikova M., Ortega-Rodriguez U., Xiang R., Phue J.-N., Shen R.-F., Rozzelle J., Kapoor N., Rabara T. (2021). SARS-CoV-2 B. 1.1. 7 (alpha) and B. 1.351 (beta) variants induce pathogenic patterns in K18-hACE2 transgenic mice distinct from early strains. Nat. Commun..

[42] Nagano T., Nakano M., Nakashima A., Onishi K., Yamao S., Enari M., Kikkawa U., Kamada S. (2016). Identification of cellular senescence-specific genes by comparative transcriptomics. Sci. Rep..

[43] Saul D., Kosinsky R.L., Atkinson E.J., Doolittle M.L., Zhang X., LeBrasseur N.K., Pignolo R.J., Robbins P.D., Niedernhofer L.J., Ikeno Y. (2022). A new gene set identifies senescent cells and predicts senescence-associated pathways across tissues. Nat. Commun..

[44] Kumari R., Jat P. (2021). Mechanisms of cellular senescence: Cell cycle arrest and senescence associated secretory phenotype. Front. Cell Dev. Biol..

[45] Xu P., Wang M., Song W.-m., Wang Q., Yuan G.-C., Sudmant P.H., Zare H., Tu Z., Orr M.E., Zhang B. (2022). The landscape of human tissue and cell type specific expression and co-regulation of senescence genes. Mol. Neurodegener..

[46] Yang R.C., Huang K., Zhang H.P., Li L., Zhang Y.F., Tan C., Chen H.C., Jin M.L., Wang X.R. (2022). SARS-CoV-2 productively infects human brain microvascular endothelial cells. J. Neuroinflamm..

[47] Wu D., Wu X., Huang J., Rao Q., Zhang Q., Zhang W. (2021). Lymphocyte subset alterations with disease severity, imaging manifestation, and delayed hospitalization in COVID-19 patients. BMC Infect. Dis..

[48] Gil-Manso S., Herrero-Quevedo D., Carbonell D., Martínez-Bonet M., Bernaldo-de-Quirós E., Kennedy-Batalla R., Gallego-Valle J., López-Esteban R., Blázquez-López E., Miguens-Blanco I. (2023). Multidimensional analysis of immune cells from COVID-19 patients identified cell subsets associated with the severity at hospital admission. PLoS Pathog..

[49] Diao B., Wang C., Tan Y., Chen X., Liu Y., Ning L., Chen L., Li M., Liu Y., Wang G. (2020). Reduction and functional exhaustion of T cells in patients with coronavirus disease 2019 (COVID-19). Front. Immunol..

[50] Peng X., Ouyang J., Isnard S., Lin J., Fombuena B., Zhu B., Routy J.-P. (2020). Sharing CD4+ T cell loss: When COVID-19 and HIV collide on immune system. Front. Immunol..

[51] Laine L., Skön M., Väisänen E., Julkunen I., Österlund P. (2022). SARS-CoV-2 variants Alpha, Beta, Delta and Omicron show a slower host cell interferon response compared to an early pandemic variant. Front. Immunol..

