# Supplementary material for: Neurobiological Alterations Induced by SARS-CoV-2: Insights from Variant-Specific Host Gene Expression Patterns in hACE2-Expressing Mice
Source: Viruses. 2025 Feb 27;17(3):329. doi: 10.3390/v17030329 (PMC11946589; doi:10.3390/v17030329)
Supplement: Supplementary file 1 [file viruses-17-00329-s001.zip › Supplementary Figure S1.pdf]

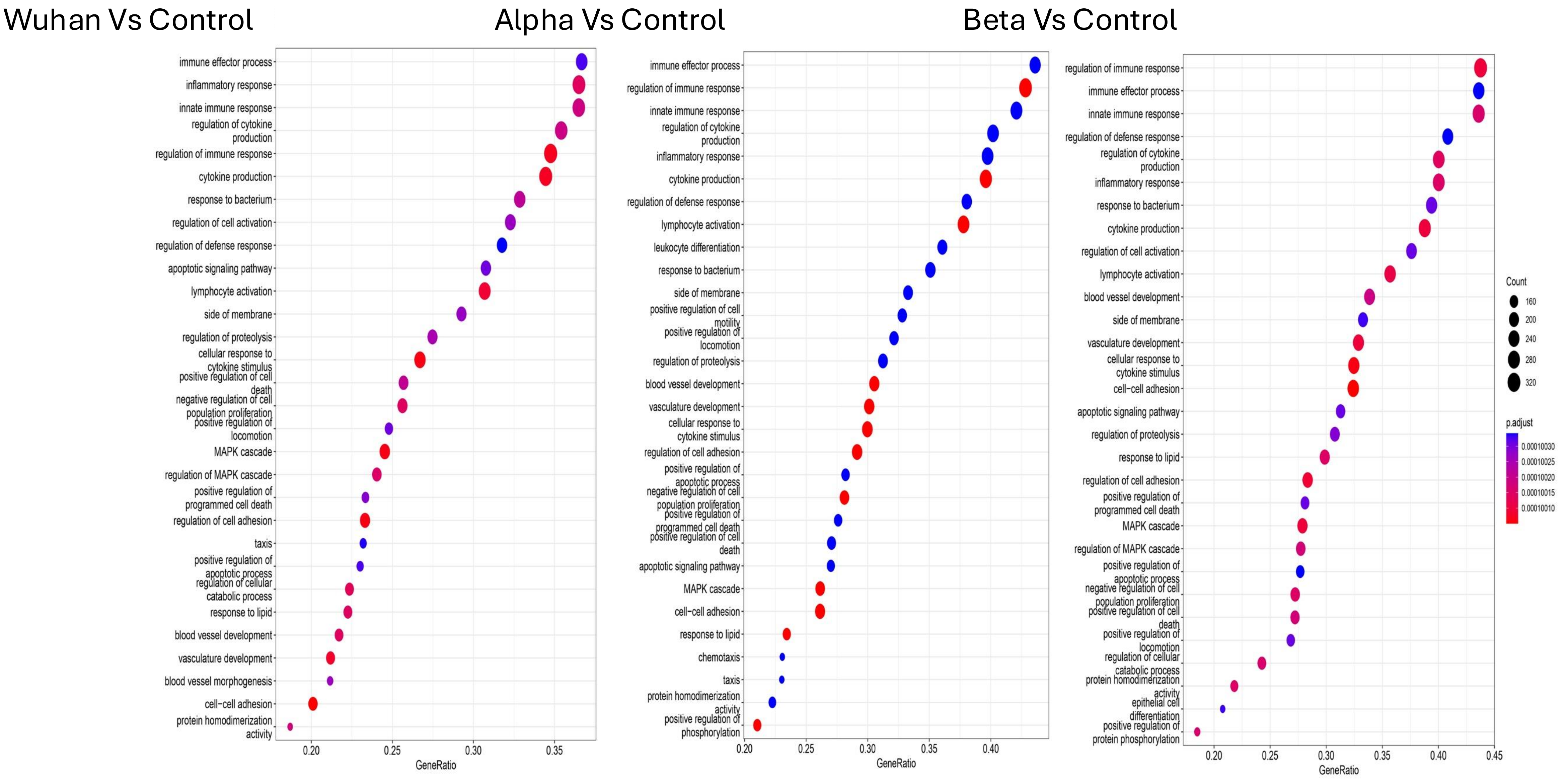

Supplementary Figure S1: GO enrichment analysis of the top 30 enriched pathways identified across the variants.

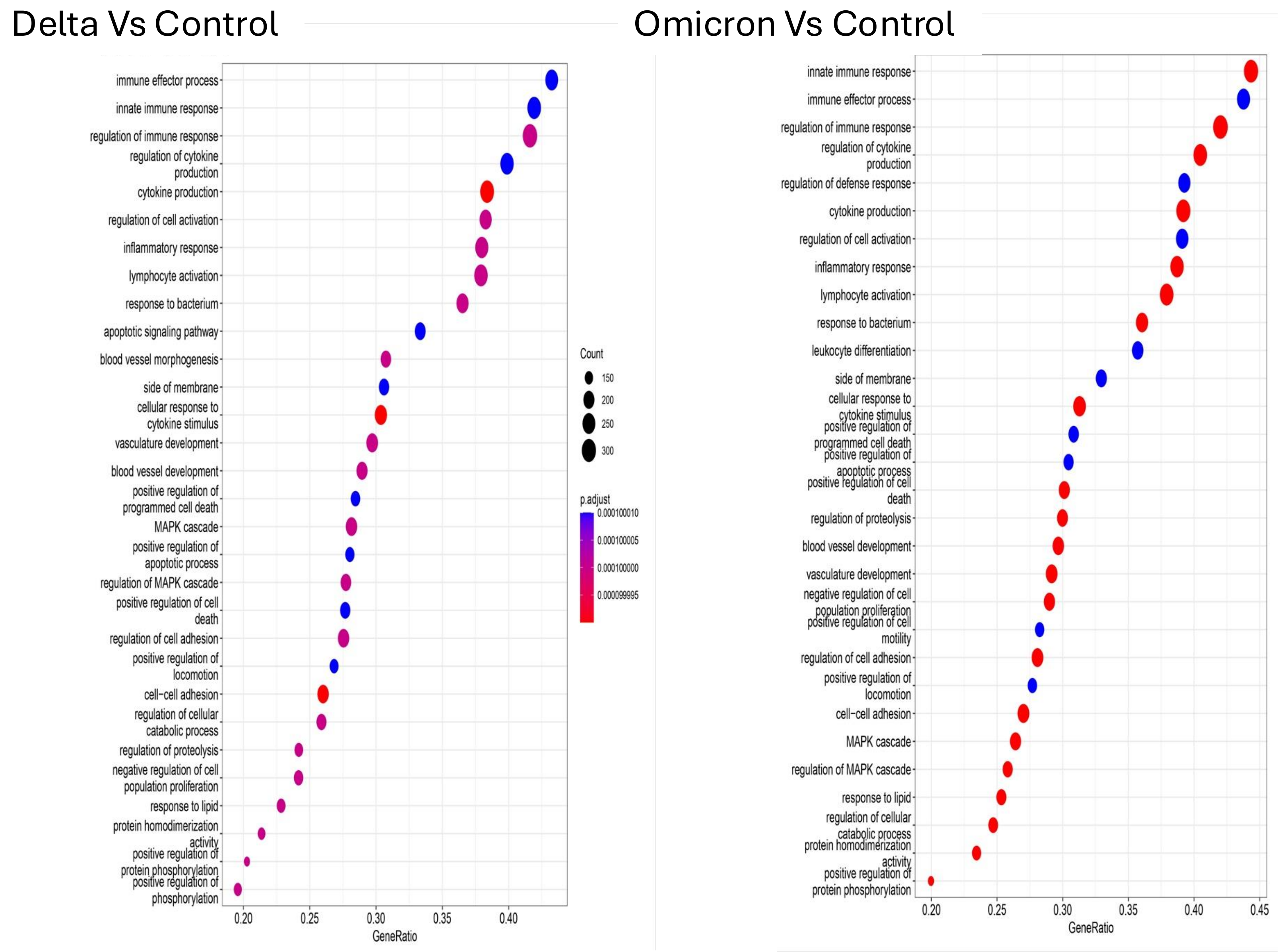

Supplementary Figure S1: GO enrichment analysis of the top 30 enriched pathways identified across the variants.
